# Supplementary material for: Molecular Characterization and Phylogenetic Analysis of Flightin Gene in Vespa basalis (Hymenoptera, Vespidae)
Source: Animals (Basel). 2024 Mar 21;14(6):978. doi: 10.3390/ani14060978 (PMC10967473; doi:10.3390/ani14060978)
Supplement: Supplementary file 1 [file animals-14-00978-s001.zip › animals-2851578-supplementary.pdf]

Table S1. Accession No

Family	Species	Gene Bank IDs
**Vespidae**	*Vespa mandarinia*	XP_035737952.1
	Vespa crabro	XP_046824901.1
	Vespa velutina	XP_047357593.1
	Vespula vulgaris	XP_050858048.1
	Vespula pensylvanica	XP_043673380.1
	Polistes dominula	XP_015177827.1
	Polistes fuscatus	XP_043495209.1
**Apidae**	*Bombus vancouverensis nearcticus*	XP_033205116.1
	subgenus *Bombus huntii*	XP_050473413.1
	Bombus impatiens	XP_003491527. 1
	Bombus terrestris	XP_003394698.1
	Bombus ignitus	*8EW5_A Chain A*
	Apis dorsata	*XP_006618153.1*
	Apis florea	XP_003696584.1
	Apis laboriosa	XP_043792750 .1
	Apis mellifera caucasica	KAG6799352.1
	Apis cerana	XP_016912210.1
	Megachile rotundata	XP_003706340.1
	Melipona quadrifasciata	KOX67587.1

	Frieseomel ittavaria	XP_043521843. 1
	Habropoda laboriosa	XP_017790128.1
	Colletes gigas	XP_043257386.1
	Osmia lignaria	XP_034180962.1
	Ceratina calcarata	XP_017884125.1
**Formicidae**	*Camponotus floridanus*	XP_011267943.1
	Monomorium pharaonis	XP_012533138.1
	Pogonomyrmex barbatus	XP_011629524.1
	Dinoponera quadriceps	XP_014479977.1
	Harpegnathos saltator	XP_011154535.1
	Odontomachus brunneus	XP_032685464.1
	Acromyrmex echinatior	XP_011064338.1
	Pseudomyrmex gracilis	XP_020284828.1
	Cataglyphis hispanica	XP_050455871.1
	Formica exsecta	XP_029672853.1
	Nylanderia fulva	XP_029155503.1
	Solenopsis invicta	XP_011171429.1
	Temnothorax longispinosus	TG Z32040.1

```
Vespa basalis                      M-WD--D-EPAPWDIE-ETAP-EE-TGDQEG--A--KEG-G-----AEGAA---PGELPKVKLEKI
Vespa mandarinia                   M-WD--D-EPAPWDIE-ETAP-EE-TGDQEG--A--KEG-G-----AEGGA---PGELPKVKLEKI
Vespa crabro                       M-WD--D-EPAPWDIE-ETAP-EE-TGDQEG--A--KEG-G-----AEGVA---PGELPKVKLEKI
Vespa velutina                     M-WD--D-EPAPWDIE-ETVP-EE-TGEQEG--A--KEG-G-----AEVVA---PGELPKVKLEKI
Vespula vulgaris                   M-WD--D-EPAPWDLD-EPAA-EE-TEAQEG--A--PEG-A-----AEGAE---PGELPKVKLEKI
Vespula pensylvanica               M-WD--D-EPAPWDLD-EPEA-EE-TQAQEG--A--PEG-A-----AEGAE---PGELPKVKLEKI
Polistes dominula                  M-WD--D-EAPAWEFE-ETPA-EQ-PA--EG--AEGAEG-AP----AE-GE---AAEKPKIKLLKL
Polistes fuscatus                  M-WE--D-DTSAWEFE-ETPA-EE-TA--EG--AEGAAA-AP----AE-GE---AAEKPKIKLEKI
Bombus vancouverensis nearcticus   M-WAD-E-EPAPWDIE-ETPA-EQ-AP--EA--S--AES-AA--P-AAEAA---TGEKPKIKLEKI
Bombus huntii                      M-WAD-E-EPAPWDVE-ETPA-EQ-AP--EA--S--AES-AA--P-AAEAA---TGEKPKIKLEKI
Bombus impatiens                   M-WAD-E-EPAPWDVE-EAPA-EQ-AP--EA--S--AES-AA--P-AADAA---TGEKPKIKLEKI
Bombus terrestris                  M-WAD-E-EAAPWDIE-ETPA-EE-TP--EA--S--AET-AA--P-AGEAA---TGEKPKIKLEKI
Bombus ignitus                     M-WAD-E-EPAPWDIE-ETPA-EQ-AP--EA--S--AES-AA--P-AAEAA---TGEKPKIKLEKI
Apis dorsata                       M-WAD-E-EPAPWDIE-ETPE-EQ-PA------A--AEG-AP--A-AEAAA---PGEKPKIKLEKI
Apis florea                        M-WAD-D-EPAPWDIE-ETPE-EQ-PA--EGE-A--AEG-AP--A-EGAAA---PGEKPKIKLEKI
Apis laboriosa                     M-WAD-E-EPAPWDIE-ETPE-EE-PE--EA--A--AEG-AP--A-AEAPA---PGEKPKIKLEKI
Apis mellifera caucasica           M-WAD-E-EPAPWDIE-ETPE-EQ-PE--EA--P--AEG-AA--A-AEAAA---PGEKPKIKLEKI
Apis cerana                        M-WAD-E-EPAPWDIE-ETPE-EQ-PE--EA--P--AEG-AP--A-AEAAA---PGEKPKIKLEKI
Melipona quadrifasciata            M-WD--D-EPAPWDLD-ETPA-EE-PA--EG--A--AEG-GTEGG-EGAAA---PAEKPKIVLQKI
Megachile rotundata                M-WD--D-EPAPWDIE-ETPE-EQ-PA--EGEAA--AEG-----G-A-AAA---PAELPKVKLEKI
Frieseomelitta varia               M-WD--D-EPAPWDVD-ETPA-EQ-PE--EG--A--AEG-AAEGGGAAAA---PAEKPKVVLLKL
Habropoda laboriosa                M-WAD-D-EPAPWDIE-ETPE-EQ-PG-EEA--A--AEG-AP--A-EGQAA---PGEKPKIKLEKI
Colletes gigas                     M-WADED-EPAPWDIE-ETPE-EQ-PA--DD-AP--AEG-AA--P-AAAAA---PGEKPKIKLEKI
Osmia lignaria                     M-WD--D-EPAPWDIE-DTPA-EE-TA--EG--A--AEG-AAEGA-APAAA---PGEKPKIKLEKI
Ceratina calcarata                 M-WD--D-EPAPWDIGDDTPA-DQ-PA-EEGA-A--AEG-AP----AEGAAAKDAAEKPKITLLKL
Camponotus floridanus              M-WD--D-EPAPWDIE-EPQP-A---A--EG--T--TEG-AP----AE-GA---APETPVLKLEKL
Monomorium pharaonis               MSWM--EEEAAPWDSE-EPAP-EA-SA--EG-----AEG-AP----AQ-PA---ATERPVLKLEKL
Pogonomyrmex barbatus              M-WE--D-EPAPWDIE-EPAP-EA-GA--EG--A--TEG-AA----TE-AA---ATEKPVLKLLKL
Dinoponera quadriceps              M-WE--D-EPAPWDIE-APAP-EGATA--EG--T--AEG-AP----VE-GA---ASEQPVLKLEKI
Odontomachus brunneus              M-WN--D-ESAPWDLE-EPTP-EA-AV--EG--A--AEG-AP----AE-GA---APEKPALKLEKI
Acromyrmex echinatior              M-WT--DEESAPWDIE-EPAP-EA-GA--EG--A--TEG-AP----VEGAA---AAEKPVLKLLKL
Cataglyphis hispanica              M-WD--D-EPAPWDLE-ETAP-DA-AV--EG--A--AEG-AP----AE-GA---APEKPVLKLEKI
Harpegnathos saltator              M-WN--D-EPASWDIE-EPAP-AA-TT--EG--A--AEG-AP----AE-GA---APERPVLKLEKI
Pseudomyrmex gracilis              M-WD--D-EPASWDIE-EPAA-A--TE--GA--T--AEGEAP----AE-GA---VSDKPVLKLEKL
Formica exsecta                    M-WD--D-EPAAWDTE-ETAP-EA-AA--EG--A--AEG-AP----AE-GA---APEKPVLKLEKI
Nylanderia fulva                   M-WD--D-EPAAWDVQ-EEAPPEA-AA--EG-----AEG-AP----AEGA----APEKPRLTLLKL
Solenopsis invicta                 M-WD--D-EPAPWDAE-EPAP--A-AA--DG--A--AEG-AP----AEGAP---ATEKPVLKLLKL
Temnothorax longispinosus          MSWA--DDEAAPWDIE-EPAA-EA-VA--EG--A--GEG-AP----AE-AA---TTDRPVLKLLKL

cons                               * *     : :...*:                                        : * : * *:

Vespa basalis                      EPPKYNHHWVRPLFLNYD-YLYDYRKNYYDDVIDYLDQRQKGLYREPPRAQEWAERTMRTYDQKNL
Vespa mandarinia                   EPPKYNHHWVRPLFLNYD-YLYDYRKNYYDDVIDYLDQRQKGLYREPPRAQEWAERTMRTYDQKNL
Vespa crabro                       EPPKYNHHWVRPLFLNYD-YLYDYRKNYYDDVIDYLDQRQKGLYREPPRAQEWAERAMRTYDQKNL
Vespa velutina                     EPPKYNHHWVRPLFLNYD-YLYDYRKNYYDDVIDYLDQRQKGLYREPPRAQEWAERTMRTYDQKNL
Vespula vulgaris                   EPPKYNHHWVRPLFLNYA-YLYDYRKNYYDDVIDYLDQRQKGLYREPPRAQEWAERAMRTYDQKNI
Vespula pensylvanica               EPPKYNHHWVRPLFLNYA-YLYDYRKNYYDDVIDYLDQRQKGLYREPPRAQEWAERAMRTYDQKNI
Polistes dominula                  EPPKYNHHWVRPLFLNYD-YLYDYRQNYYNDVIDYLNQRARGLFREPPRAQEWAERALRMYDAKNI
Polistes fuscatus                  EPPKYNHHWVRPLFLNYD-YLYDYRQNYYNDVIDYLNQRARGVFREPPRAQEWAERALRMYDTKNI
Bombus vancouverensis nearcticus   EPPHYNHHWVRPLFLNYA-YLYEYRKNYYNDVIDYLNQREKGIFREPPRAQEWAERAMRTYDEKNT
Bombus huntii                      EPPHYNHHWVRPLFLNYA-YLYEYRKNYYNDVIDYLNQREKGIFREPPRAQEWAERAMRTYDEKNT
Bombus impatiens                   EPPHYNHHWVRPLFLNYA-YLYEYRKNYYNDVIDYLNQREKGIFREPPRAQEWAERAMRTYDEKNT
Bombus terrestris                  EPPHYNHHWVRPLFLNYA-YLYEYRKNYYNDVIDYLNQREKGIFREPPRAQEWAERAMRTYDEKNT
Bombus ignitus                     EPPHYNHHWVRPLFLNYAYYLYEYRKNYYNDVIDYLNQREKGIFREPPRAQEWAERAMRTYDEKNT
Apis dorsata                       EPPHYNHHWVRPLFLNYA-YLYEYRKNYYDDVINYLNQRQKGLFREPPRAQEWAERAMRTYDEKNL
Apis florea                        EPPHYNHHWVRPLFLNYA-YLYEYRKNYYDDVINYLNQRQKGLFREPPRAQEWAERAMRTYDEKNV
Apis laboriosa                     EPPHYNHHWVRPLFLNYA-YLYEYRKNYYDDVINYLNQRQKGLFREPPRAQEWAERAMRTYDEKNV
Apis mellifera caucasica           EPPHYNHHWVRPLFLNYA-YLYEYRKNYYDDVINYLNQRQKGLFREPPRAQEWAERAMRTYDEKNV
Apis cerana                        EPPHYNHHWVRPLFLNYA-YLYEYRKNYYDDVINYLNKRQKGLFREPPRAQEWAERAMRTYDEKNV
Melipona quadrifasciata            DPPHYNHHWVRPLFLNYD-YIYEYRKNYYDDVIDYLNQRQKGIYREPPRAQEWAERAMRTYDQKNT
Megachile rotundata                EPPHYNHHWVRPLFLNYA-YLYEYRKNYYDDVIDYLNQRQRGIFREPPRAQEWAERVMRTYDEKNT
Frieseomelitta varia               EPPHYNHHWVRPLFLNYD-YLYEYRKNYYDDYIDYLNQRQKGIFREPPRAQEWAERAMRTYDQKNT
Habropoda laboriosa                DPPHYNHHWVRPLFLNYA-YLYEYRKNYYDDVIDYLNRRQKGLFREPPRAQEWAERAMRTYDMKNT
Colletes gigas                     EPPHYNHHWVRPLFLNYA-YLYDYRKNYYDDVIDYLNQRQRGIFREPPRAQEWAERAMRTYDEKNT
Osmia lignaria                     EPPHYNHHWVRPLFLNYD-YIYEYRKNYYDDVIDYLNQRQRGIFREPPRAQEWAERAMRTYDEMNT
Ceratina calcarata                 EPPHYNHHWVRPLFLNYA-YLYDYRQNYYNDVIDYLNQRQKGLFREPPRAQEWAERAMRTYDQKNI
Camponotus floridanus              EPPHYNHHWVRPLFLNYD-YLYDYRKNYYDDVIDYLNQRQKGLFREPPRAQEWAERVLRTYDQKNI
Monomorium pharaonis               EPPHYNHHWVRPLFLNYA-YLYDYRKNYYDDVIDYLDSRQKGIFREPPRAQEWAERTMRTYDQKNV
Pogonomyrmex barbatus              EPPHYNHHWVRPLFLNYA-YLYDYRKNYYDDVIDYLDSRQKGIFREPPRPQEWAERALRTYDQKNI
Dinoponera quadriceps              EPPHYNHHWVRPLFLNYE-YLYDYRKNYYDDVIDYLNQRQRGMYREPPRAQEWAERALRTYDQKNM
Odontomachus brunneus              EPPHYNHHWVRPLFLNYA-YLYDYRQNYYNDVIDYLNQRQKGIYREPPRAQEWAERAIRTYDQKNE
Acromyrmex echinatior              EPPHYNHHWVRPLFLNYA-YLYDYRKNYYDDVIDYLNSRQKGIYREPPRAQEWAERAIRTYDQKNM
Cataglyphis hispanica              EPPHYNHHWVRPLFLNYA-YLYDYRKNYYDDVIDYLNQRQKGIFREPPRAQEWAERTLRTYDQKNK
Harpegnathos saltator              EPPYYNHHWVRPLFLNYA-YLYDYRKNYYNDVIDYLNHRQKGLFREPPRAQEWAERAMRTYDQKNM
Pseudomyrmex gracilis              EPPHYNHHWVRPLFLNYT-YLYNYRKNYYDDVIDYLNQRQKGLYREPPRAQEWAERAMRTYDQKNK
Formica exsecta                    EPPHYNHHWVRPLFLNYA-YLYDYRKNYYDDVIDYLNQRQKGIFREPPRAQEWAERTMRIYDQKNK
Nylanderia fulva                   EPPHYNHHWVRPLFLNYD-YLYDYRKNYYDDVIDYLNHRQKGLFREPPRAQEWAERAMRTYDQKNT
Solenopsis invicta                 EPPHYNHHWVRPLFLNYA-YLYDYRKNYYNDVIDYLDSRQKGIYREPPRAQEWAERAMRTYDEKNV
Temnothorax longispinosus          EPPHYNHHWVRPLFLNYA-YLYDYRKNYYDDVIDYLDSRQKGIYREPPRAQEWAERALRTYDEKNI

cons                               :** *************  *:*:**:***:* *:**: * :*::*****.******.:* **  *
```

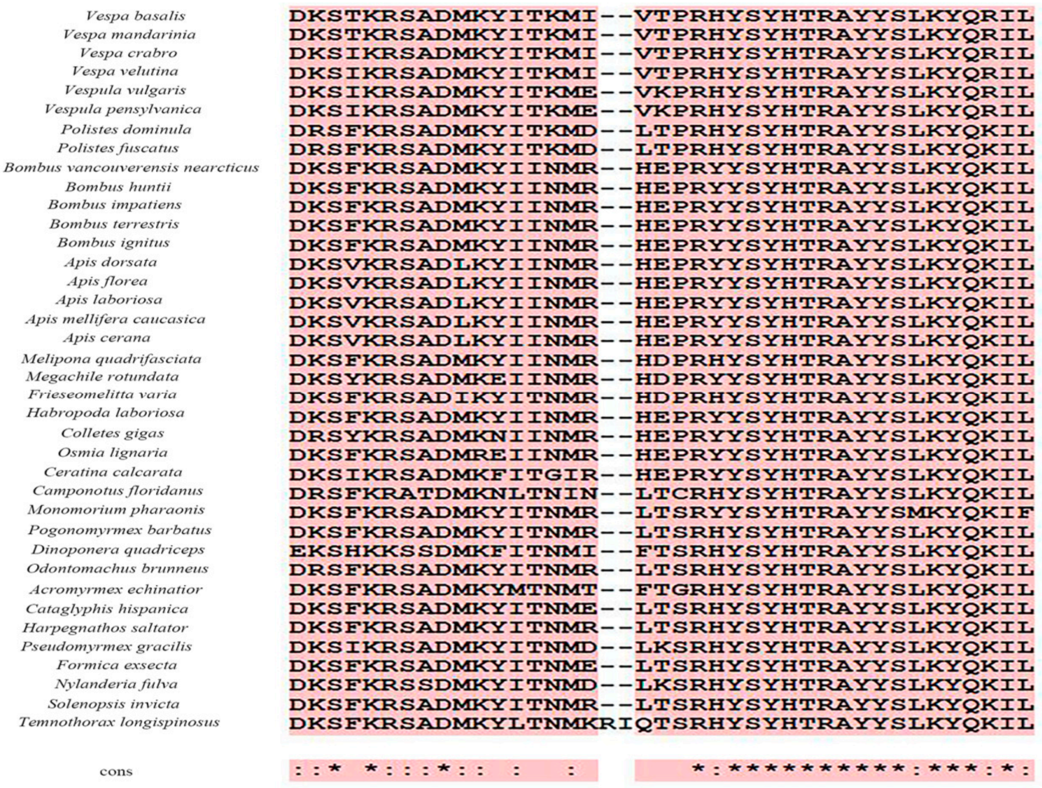

Figure S1. Protein Homology

Table S2. Primers details

primer name	Primer No	primer sequence 5-3
blue.fasta-1		
RT1569-A-P1F	P1	*CATTCGATCTGTTTGATATAAAAGAGC*
RT1569-A-P1R		*CTTTAGCGATTCTAACTTCGACACT*
RT1569-A-P2F	P2	*GGGATACCTAACTACGTGCAGTTTC*
RT1569-A-P2R		*TCCATTTGATGCTTGCTTTATTC*
ul.fasta-1		
RT1569-C-P1F	P3	*ATCTTTTAAAAAAGAACCGATTTGTTG*
RT1569-C-P1R		*GGTATCGGTTAAATAATCAAAAGAACAG*
RT1569-C-P2F	P4	*ATGGAAAATTATCACGTGGTCAAGT*
RT1569-C-P2R		*TTATATGCCAGGAATTTACTTTATTGG*
Vespa velutina flightin (LOC124952177), mRNA(1).fasta-1		
RT1569-D-2F	P4	*ATAAAAGCAAGAAAGAAGGCAAGGT*
RT1569-D-2R		*TATCGGCGGATCTCTTAATACTCTT*
RT1569-D-P2F	P5	*CGCCCATTATTTCTCAATTATGACT*
RT1569-D-P2R		*TAAGTAAAAGTGCACGTGTATTTTATAAT*

S5. full length of flightin

identification number	sequence
**blue.fasta-1**	CATTCGATCTGTTTGATATAAAAGAGCTCACTAACTTTGGTACGGGTCAAGTGAGTTGCGGAACGTGCT<br>GTCAACTTATCGCACCGCGTCTCACTTCAATTCAGATTCGAACGGACGTAGAAATAAAGTAGAAGTTTA<br>TTTTACATCTTAACTTGACATATCTATTCAAGGTCCTAAGGGTTGAAACCTTTTGGACGTGGATAGGACG<br>AGTCAAGACGACCACTAAAACTCTTCTTCGTTCAGACATCTCGTTTATCTCGGAAGATCCATTTTAACGA<br>TTCGTCAGTCTACCAACCGAGGACACAGCCTTGAAGAAGGATGCTGCTGCAAAACTATACCTTCGAGA<br>ACGGCCCTCAGCCTTTTGTAGGCGGAATGGGAGAGGTCCAACAAATGCAAGAAAGATTTTTGGGATG<br>GAACGTACCACCGGAACACATGGGACTCGTACACGTTCATTGGAGGGGCTACCTAGCACCTGGAAAAT<br>ACTGGCACATTGGTCTCTCGCTTATTTATTTTATGCTGTTTGTCATGTCCGTCGTTGGAAATGGATGCGTC<br>GTATGGATATTTAGCACATCAAAAGCTCTTAGGACACCCTCGAACATGTTTATCGTCAATTTGGCTATATT<br>CGATCTAACGATGGCGTTAGAAATTCCACATTTCATAATAAATAGCTTTATACAACGCATTTGGGGTTGG<br>GAGATTGGTTGTGCTATATTTGCATTTTTGGGCAGTATTTCTGGTATCGGGCAGGCAATCTCCAACGTTG<br>CCATTGCCTACGATCGTTACAGAACGATATCGTGTCCGATCGATGGTAGACTAAACGGATCGCAAGCTG<br>GATTGATCGTTGCATTTACTTGGTTCTGGGCTTTACCATTTTCAGTTTTACCTCTTATAGAGGTATGGGG<br>AAAATTTGTACCAGAGGGATACCTAACTACATGCAGTTTCGATTTTCTAACGGATAACTCGGATACGAG<br>GGTTTTCGTAGCTTCGATCTTTCTTTGGTCTTATCTTTTACCATTGACCCTCATAGTTTATTTTTACTCGCA<br>ATTGATTAGATCCATTAGACAACACGAGAAGATGCTTCGCGATCAGGCAAAAAAAATGAACGTGAAAT<br>CTTTGGTTTCAAATCAGGATAAAGAACGTAGCGTCGAAGTTAGAATCGCTAAAGTAGCGTTCACTATTT<br>TCTTCTTATTTCTCTGCGCGTGGACGCCTTATGCTGTTGTCGCTGTTATCGGTGCTTTTGGTAATAGCGA<br>CTTGTTGACACCTCACGTAGGTATGTTACCAGCAGTTTTCGCAAAATCGGTTTCGTGCATAGATCCATGG<br>ATTTATGCCATCAATCATCCAAGGTATCGTCAGGAATTACAAAAGCGATGCAAGTGGATGGGAATACGA<br>GAACCCGAACCAGTTGCAGACAATGTTTCAGCGCAGACTGAAAAACTTAAGGCAGACGAAGCATAAT<br>TCTAAATGTTTTATGTACTTTATGGAAAATGAAAAAACGACGGCATGGATGATCGATCGTATCGAATCGT<br>CAATCCACCACCGATTCGACATCGATCTTTTGTCTCGTCTACGATTTCTTTTATTTGTCAATATATTATAAAT<br>AGAAATTATTACCAAAATATTCTGTATAAAAATTGTCGTTATTATTATTATTATTATTATTATTATTATTAT<br>TCTTATTATTATTATTATTATCATTATACAGTAGAACGTGGCACATACACGCTAATCTGTGGTTTCTTATATA<br>AATTTGAATAAAGCAAGCATCAAATGGA
**ul.fasta-1**	ATCTTTTAAAAAAGAACCGATTTGTTGGATTAAATTTCTACTTTTGTTCCATACAAAAATGGATACTAA<br>AAATGGAACTAAATCGACACAAAATAAGATACTATGAAAAAGTAGTCAACAGCAATGAAGATAAGA<br>ATGTATACTATAATTCGAAATCGATTGATTTGACTTTGAAGTTTGCTCTTGCTGACCAACGGTAATCA<br>AGGGATGATCCGTAGCTGATCCTCAAATGAAGCCATATCGTCGAGCTGATTAAAGGTTTCATGGATT<br>TAATTTGACAGATATGGTATAAAAGGCCACATCTTGGTTAATTCAAAAACAAGGTGAGGGACTGGTA<br>GACGGTGACCACCAACATGTTCAACGACAGTATTCATTGGGAGGCGAGAGTTCTACCAGCAGGTCC<br>ACCACGCCTATTGGGATGGAACGTCCCTGCCGAAGAATTGGTACATGTACCGGAACATTGGCTGGTC<br>TTTCCTGAACCCAATCCCTCCCTTCATTATATGCTGGCCCTGTTATATATATTATTCACATTCATTGCTT<br>TACTTGGCAATGGTCTCGTCATATGGATATTTTGCGCTGCCAAATCCTTGAGGACTCCCTCGAACATG<br>TTCGTTGTGAATCTTGCGATTTGCGATTTTGCCATGATGATGAAGACACCAATTTTTATATACAATTCA<br>TTTAACACCGGCTTTGCTTTGGGCAATCTGGCATGCCAGATTTTTGCTACGATTGGTTCATTGTCTGG<br>CATTGGTGCAGCGGTAACTAATGCTGCGATAGCATATGATAGATACAGTACCATAGCCAGACCATTG<br>GATGGAAAATTATCACGTGGTCAAGTGATTCTCTTTGTCGGATTAATTTGGGTTTACGTTTTGCCTTG<br>GGTATTAATGCCTGCAATGGGTGTTTGGGGTCGTTATGTACCTGAAGGATTTCTTACAAGCTGTTCTT<br>TTGATTATTTAACCGATACCGCTGAAACAAGATATTTCGTTTTAACTTTATTTATTTTCTCCTATTGTTT<br>ACCAATGTCACTTATTGTATATTATTACAGTCAAATTGTTAGTCATGTTGTTAATCACGAAAAGGCTCT<br>TCGAGAACAAGCAAAGAAGATGAACGTTGAAAGTTTACGAAGTAACGCCAATCAGAATGCTCAATC<br>TGCTGAAATTCGTATAGCGAAGGCTGCCATTACGATTTGTTTCCTATTTGTTGCCGCATGGACACCAT<br>ACGCAGTACTTGCTATGATCGGTGCATTTGGAAACAAAGCGTTGCTTACTCCCGGCGTTACTATGATC<br>CCTGCATGCTTTTGCAAAAGTGTTGCCTGTATTGATCCATATGTTTATGCGATCAGTCATCCAAAATA<br>CAGACTCGAGTTGCAAAAACGATTACCATGGTTAGAGCTTCAAGAGAAACCAATCGCATCCGACAGT<br>GCAAGTACAACTACCGAAACTGTAAATACAGCACCACCAGCTTCGAGTTAATCTACTTCTACATTTGA<br>CACAATAACGGATCGATGATAAAAAAAAAATGAAAAAAGTACGCTCGTAATACTCCGATTGTATGAT<br>TGCCTTCACTGTAACTCAAAATATGTGATTATATTCTTGTATAGTAAATGACATTCCAATAAAGTAAAT<br>TCCTGGCATATAA

**Vespa velutina flightin (LOC124952177), mRNA(1).fasta-1 partial experimental sequence**	ATAAAAGCAAGAAAGAAGGCAAGGTCCCCGCGAGTGGCGAAGCTTCAAAGACGAGAGGGTAGTTT<br>TTTTTGCAGTCTGTATAGGTGCTTCGTAGAGCGAGCATCGTAGCAAAGGCTTTCTAAAGAGAATTGA<br>AGAAAAAGAAGAAGAAGTAAAAGAAGGATGTGGGACGATGAACCAGCTCCGTGGGATATCGAAG<br>AAACGGCTCCCGAAGAAACAGGAGACCAAGAAGGAGCCAAGGAAGGGGGAGCTGAAGGGGCTGC<br>ACCCGGTGAACTACCAAAAGTAAAGCTCGAAAAGATCGAACCTCCAAAGTACAATCATCATTGGGTG<br>CGCCCATTATTTCTCAATTATGACTATCTTTACGATTACAGGAAGAATTATTACGACGACGTAATCGA<br>CTATCTCGATCAAAGACAAAAGGGTTTGTATCGTGAGCCTCCAAGAGCACAGGAATGGGCAGAAAG<br>GACCATGAGGACGTACGATCAGAAGAACCTTGATAAGAGTACTAAGAGATCCGCCGATATGAAATA<br>CATCACTAAAATGATAGTTACGCCAAGACATTATAGCTATCATACGCGGGCATATTATAGCTTGAAGT<br>ACCAGAGAATTCTTTGAGTTATCATTTTTGATGGAGAATGATCGGCGAAGATTGACGATTAAAGATC<br>TTACACTATTCTTCGTCGATATTGCTATTACCGATAATGCCAGTGGTAATCCTTTTTTTAATCGGCTTA<br>CCTATACGTTGTATATAACATCATCTTAGACATGAGGATGTTGTATTTTAAAAATAAATCGTAGTCGT<br>CGTAGATATTTATTCTGGATCTTTCATTAAACGAGGGAAAAGATCACGGTTTACCTCTGGCCAAATTT<br>ATTTATCGAGTAGCACTTATCGTCTAATGTATCGATAATTTCATTTCTAGATAAATTCGATTCGATTTC<br>ATACGCGATTCATTTATCTTCGTAGATATATATTTTTCATAAATAGGGACAAACATTCGCATAGAACTT<br>CGATATCTTATCCCATCAACAAATCAATCGTCATAAATTTAAACGCGTACTCGTTTTATTTGTTTCGTTT<br>GATTATTTATTTGTCATCAATTTTATGATATAGCTATGACGTGCTTATGTACTATGCCTATTGTATATG<br>TATTACTGTTATAAATCGGTATTATAAAATACACGTGCACTTTTACTTA
